# The effect of co-regulation on English public speaking self-efficacy in collaborative oral presentations

**DOI:** 10.3389/fpsyg.2024.1423607

**Published:** 2024-09-02

**Authors:** Xia Hao, Hua Chen

**Affiliations:** School of Foreign Studies, Nanjing University, Nanjing, China

**Keywords:** self-efficacy, co-regulation, oral presentation, collaboration, English public speaking

## Abstract

This study investigated the influence of co-regulation on public speaking self-efficacy in the context of collaborative oral presentations. A total of 237 students enrolled in an English course at a university in China took part in the research. The factor analysis findings revealed that learners’ co-regulation in public speaking encompass five components: co-planning, co-monitoring, co-evaluation, effort regulation, and help-seeking. Public speaking self-efficacy, on the other hand, pertains to learners’ confidence in aspects including the topic, language use, organization, and delivery during public speaking engagements. The path analysis demonstrated that co-planning was a significant predictor of students’ self-efficacy in terms of the topic and organization. Moreover, the co-monitoring strategy exhibited direct and positive correlations with language and topic self-efficacy. Similarly, the co-evaluation strategy showed direct and positive relationships with language, delivery, and organization self-efficacy. Furthermore, both effort regulation and help-seeking strategies were found to have direct and positive impacts on organization self-efficacy. This study offers valuable implications for educators, trainers, and individuals aiming to enhance their public speaking self-efficacy in collaborative environments.

## Introduction

Learning is socially constructed and regulated through interactions in group work (Vygotsky, 1978). In the context of English as a foreign language (EFL) learning, co-regulatory group work offers an optimal framework that facilitates students’ acquisition of crucial access to academic discourses (Deng et al., 2024). One prominent form of co-regulatory group work in EFL speaking instruction is collaborative oral presentations, largely due to their enhancement of teaching and learning efficiency in large-size classes (Chou, 2011).

Research indicates that collaborative oral presentations can help reduce EFL students’ public speaking anxiety associated with delivering presentations (Kelsen, 2019), a challenge recognized as particularly significant for individual students (Woodrow, 2006). However, it is essential to recognize that the mere grouping of students together does not automatically ensure effective collaboration; students need to possess the knowledge and skills required to co-regulate their learning and collaboration (Barron, 2003; Chou, 2011). The co-regulation strategies employed during collaborative work play a reciprocal role in aiding students’ development into independent learners (Chan, 2012). Similarly, the application of co-regulation strategies by students in collaborative oral presentations should act as a foundation for nurturing positive psychological outcomes, such as task-related self-efficacy (Dörnyei and Malderez, 1997).

Given the potential impact of co-regulation strategies on enhancing students’ self-efficacy in public speaking, it is imperative to explore students’ utilization of such strategies to gain deeper insights into collaborative learning within the realm of EFL education (Qiu and Lee, 2020; Wang, 2022). This necessity becomes particularly pressing in light of observations indicating that students face challenges in grasping and summarizing key ideas during collaborative oral presentations, and struggle to effectively navigate negotiation, communication, and interactions among group members (Chou, 2011). Despite this identified research gap, there remains a dearth of studies in this domain, resulting in the benefits and challenges associated with employing co-regulation strategies to enhance public speaking self-efficacy remaining largely uncharted.

## Literature review

### Collaborative oral presentation

Collaboration is defined as “a coordinated, synchronous activity that is the result of a continued attempt to construct and maintain a shared conception of a problem” (Roschelle and Teasley, 1995, p. 70). Central to the definition is “a shared conception of the problem” where participants need to have social interactions and co-construct knowledge in a joint problem space. Collaborative oral presentations offer students the opportunity to engage in such an environment for tackling authentic challenges within speaking exercises.

To successfully execute a collaborative oral presentation, students engage in a collective effort to enhance negotiability, interactivity, and dialogic exchanges within their collaborative discourse interactions (Reusser, 2001). This entails collaborative activities such as jointly developing and refining the presentation, offering feedback to elevate linguistic competence and presentation skills, leveraging technology to enhance technical proficiency and visual aesthetics of presentations, as well as providing reciprocal reminders of deadlines and strategic support to combat procrastination (Nguyen, 2013). In the collaborative task, participants amalgamate objectives, characteristics of the existing problem scenario, understanding of potential problem-solving strategies, and actionable steps.

Peer scaffolding is a defining feature of collaborative oral presentations, with students engaging in six distinct categories of behaviors, including workload distribution, idea and resource pooling, technological assistance, peer feedback, audience interaction support, and emotional assistance. Students partake in preparatory tasks beyond the classroom setting by utilizing peer dialogues to delineate task specifications, brainstorm ideas, seek peer comments, provide rehearsal coaching, and deliberate on slide composition (Yang, 2010).

Collaborative group work has the potential to offer support and assistance during challenging periods in the language learning process (Dörnyei and Malderez, 1997). Collaborative oral presentations have been shown to enhance students’ involvement (Barry, 2012) and motivation (Al-Issa and Al-Qubtan, 2010) within the learning activity.

Nevertheless, although it is suggested that collaborative group work leads to greater use of communicative strategies and benefits oral presentation ability (Kelsen and Liang, 2019), problems remain. As indicated by Chou (2011) in her examination of the challenges associated with group work, collaborative oral presentations may encounter issues related to intra-group coordination if not managed effectively. However, it is indicated that this risk can be mitigated through co-regulation (Volet et al., 2009).

### Co-regulation

Co-regulation is defined as the mechanism through which social contexts provide assistance or frameworks for individual engagement and educational advancement, with clusters of individuals functioning as different self-regulating entities who oversee one another’s learning or task completion in a social setting (Volet et al., 2009). Enabling co-regulation within a collective entails employing various tactics, such as collaboratively devising shared educational objectives, collectively supervising the learning procedures, and jointly assessing the group’s final outcome (Ucan and Webb, 2015). In the process of co-regulation, the regulatory focus encompasses content supervision, task comprehension, time management, emotion control, and organizational management (Li et al., 2021).

In the context of power distribution, co-regulation strategies span from “individual regulation within the group,” in which one individual temporarily assumes leadership in regulating the learning activity within the group, to “co-regulation as a group,” where all group members consistently engage in monitoring and regulating the joint activity (Volet et al., 2009). The regulatory role assumed within the group may also involve temporary control over specific task segments (Salonen et al., 2005).

Co-regulation is conceptualized within the framework of five components: co-planning, involving collaborative goal setting, task division, and planning by students; co-monitoring, referring to students’ continuous management of their understanding, progress, and performance; co-evaluating, focusing on how students evaluate their group’s performance; effort regulation, concerning students’ perseverance in the face of learning obstacles or challenges; and help seeking, detailing the effort of seeking help from other students to conquer difficulties (Su et al., 2023).

Although scholarly literature posits that co-regulation can potentially mitigate the tendency to overlook the interconnectedness of engagement, participation, and knowledge development within effective collaborative learning environments (Volet et al., 2009), empirical research in this field remains limited, particularly within the context of language education. There is a notable dearth of studies focusing on the co-regulation processes of language learners (Li et al., 2021; Su et al., 2023).

### English public speaking self-efficacy

Self-efficacy is the “beliefs in one’s capabilities to organize and execute the courses of action required to produce given attainments” (Bandura, 1997, p. 3). It serves as a central and pervasive mechanism of personal perception of one’s own capabilities to exercise control over particular events.

Due to its crucial role in alleviating English speaking anxiety among EFL learners (Mede and Karaırmak, 2017), speaking self-efficacy has garnered increased attention in EFL contexts, particularly in the realm of speaking English in public settings. English public speaking self-efficacy pertains to individuals’ confidence in their capability to deliver successful English public speeches (Zhang et al., 2019; Zhang et al., 2020). The beliefs of EFL learners regarding their English public speaking capabilities have a direct impact on their actual performance in this domain. In a study by Zhang and Ardasheva (2019), a scale was developed to assess the construct of English public speaking self-efficacy among college-level EFL students, comprising four competences in public speaking, i.e., the competence in topic, organization, language, and delivery. The hierarchical structure of the four-competence model of English public speaking self-efficacy was validated rigorously through exploratory and confirmatory factor analyses involving 203 EFL learners (Zhang et al., 2023). However, owing to the limited number of studies in the realm of English public speaking, academics in this field argue for additional research to investigate the efficacy of various sources, types, and modes in English public speaking (Zhang et al., 2020).

### Co-regulation and English public speaking self-efficacy

Co-regulation is intricately linked to students’ self-efficacy in EFL learning (Shehadeh, 2011; Su et al., 2023; Wang, 2022). Through the practice of co-regulation, students participate in the formulation of questions, adopt a cautious attitude, accumulate foundational knowledge, and foster positive affect (Volet et al., 2009).

In the realm of English public speaking, verbal feedback, identified as a profound co-evaluation strategy, stands out as a pivotal source of self-efficacy (Zhang et al., 2020). Empirical investigations focusing on EFL oral presentations consistently underscore the strong association between regulatory strategies and students’ self-efficacy in public speaking. In a study involving third-year EFL students from Vietnam, Nguyen (2013) delved into peer modeling behaviors as a form of co-regulation during collaborative oral presentations. By scrutinizing reflective reports and interviews, the researcher observed that the emotional reinforcement students receive while working collectively enhances their self-efficacy in public speaking, particularly when they are delivering the presentation on stage. A recent study on English presentations has also confirmed the role of group work in providing mutual assistance, social support, and feedback when the group regulates their work, which in turn impacts speakers’ self-efficacy in public speaking (Hartono et al., 2023).

Despite existing evidence linking co-regulation strategies to EFL self-efficacy, several gaps persist. Firstly, qualitative research predominates, lacking individual assessments of components relating to co-regulation and the dimensions of self-efficacy (e.g., Nguyen, 2013; Shehadeh, 2011; Wang, 2022). It is worth mentioning that while co-evaluation has been identified as a strategy of co-regulation that is associated with English public speaking self-efficacy (Zhang et al., 2020), the effectiveness of the other three strategies remains largely unexplored in this domain. Secondly, within the limited body of studies, there is a notable imbalance that favors research on EFL collaborative writing (e.g., Qiu and Lee, 2020; Su et al., 2023) and reading (e.g., Li et al., 2021) over speaking. Lastly, the conceptualization of self-efficacy varies depending on the language skill and research context. It remains uncertain whether different dimensions of public speaking self-efficacy can still be influenced by co-regulation strategies.

### The current study

To address the above-mentioned research gap in the relationship between co-regulation and English public speaking self-efficacy in context of collaborative oral presentations, the present study was designed to investigate the structural relationships between the components of learners’ co-regulation and their self-assessed efficacy in English public speaking. To achieve this goal, the study poses two specific research inquiries:

1. What are the components of learners’ co-regulation and English public speaking self-efficacy during the collaborative oral presentation?
2. What is the relationship between the components of learners’ co-regulation and the dimensions of English public speaking self-efficacy within the context of collaborative oral presentations?

**Figure 1:**
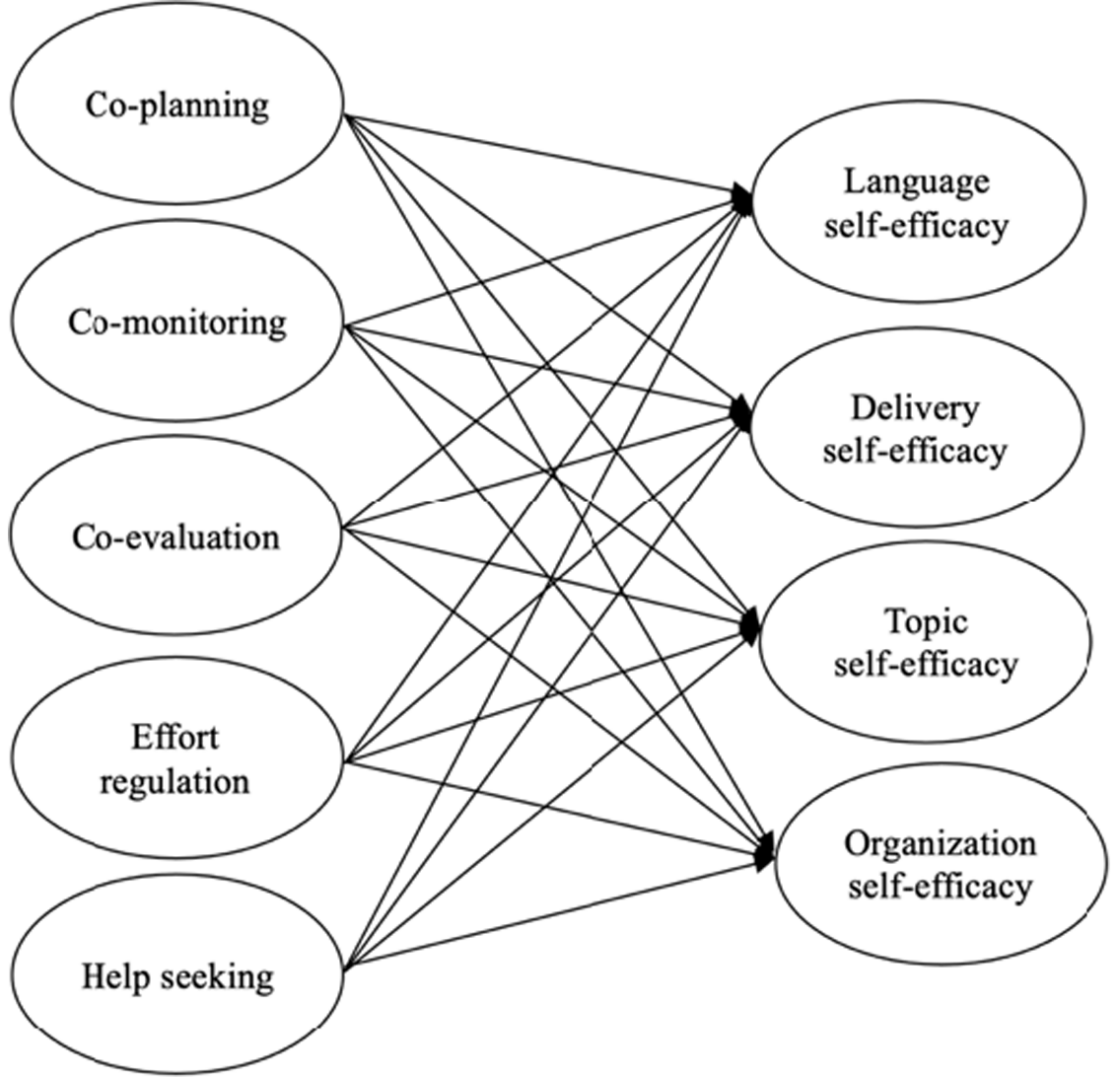
The hypothesized model of the effects co-regulation strategies on English public speaking self-efficacy.

A hypothesized research model is put forward. Considering the strong correlation between regulation strategies of EFL learners and their self-efficacy in public speaking (Nguyen, 2013; Zhang et al., 2020), it is suggested that learners’ co-regulation during collaborative oral presentations positively influence their English public speaking self-efficacy across the components. Figure 1 depicts the proposed connections between the hypothesized components of learners’ co-regulation and their self-efficacy in English public speaking.

## Research method

### Participants

Out of the total of 268 first-year undergraduate students registered in a 16-week English course, 237 students (*M*_age_ = 18.8, *SD* = 0.96; 61% male) from four intact classes consented to partake in the research and satisfactorily filled out the questionnaires of their co-regulation and self-efficacy. Prior to their involvement, the participants received detailed information regarding the objectives of the study. Assurance of confidentiality were provided at the beginning of the delivery of the questionnaires.

### Learning context

Students registered in the course were mandated to deliver presentations as a partial requirement of their academic obligations during the fall semester of the academic year 2023–2024. The collaborative group work involved the execution of oral presentations in a specified format (Kelsen, 2019). To be precise, working in teams comprising 3 to 4 individuals, students engaged in collaborative oral presentations lasting between 10 to 15 min. Every student was expected to participate in both the preparation and delivery of the presentation. In the presentation phase, every student was given 3 to 5 min to complete their section in the collaborative oral presentation.

Throughout the term, the presentation groups delivered two oral presentations. For the initial presentation, seven topics were assigned to the participants based on the course’s module topics, encompassing challenges in college life, true love, friendship, sustainable living, studying abroad, enhancing cross-cultural confidence, and fostering mutual benefits. Each group was allocated one topic for presentation. In their subsequent presentation, all groups presented their findings on the theme of the power of language at the term’s conclusion. For feedback on their presentations, each group received evaluations from their peers and assessments from the instructor, aligned with the criteria for evaluating EFL oral presentations (Wan, 2013). Apart from the questionnaire ratings, the group also received immediate verbal feedback from the instructor after their performance. Both the group as a whole and individual members were evaluated by the instructor.

### Measures

The evaluation of co-regulation strategies was conducted using the Co-Regulation Strategies (CRS) questionnaire, as devised by Su et al. (2023), which encompasses five components: co-planning, co-monitoring, co-evaluating, effort regulation, and help seeking.

The initial CRS questionnaire demonstrated strong reliability, with Cronbach’s alpha (α) coefficients of the individual components being 0.71 to 0.81 and an overall reliability coefficient of 0.85. Due to its original application in exploring students’ co-regulation strategies within computer-mediated collaborative writing tasks, adjustments were made to adapt it for evaluating co-regulation among language learners engaged in collaborative oral presentations. Specifically, one item related to online group discussions in the effort regulation was removed, and the questionnaire items were refined to suit the context of collaborative oral presentations. Instances such as “when working in our English writing group” were revised to “when working in our English presentation group.” These modifications ensured that the questionnaire aligned more effectively with the collaborative oral presentation scenarios under investigation. Each component comprises three items, resulting in a total of 15 items presented in a 5-point Likert scale format (see Appendix A).

The assessment of English public speaking self-efficacy was carried out utilizing the English Public Speaking Self-Efficacy (EPSSE) questionnaire created by Zhang and Ardasheva (2019). The original English public speaking questionnaire demonstrated high reliability, with an overall alpha coefficient of 0.87. This questionnaire comprises four dimensions: topic self-efficacy, reflecting confidence in the ability to select and maintain a topic effectively; language self-efficacy, indicating confidence in linguistic accuracy and fluency; organization self-efficacy, representing confidence in logical and clear sequencing; and delivery self-efficacy, signifying confidence in managing emotional states and physical behaviors (see Appendix B).

### Data collection and statistical analysis

The questionnaires were distributed to the students by the instructor upon completion of the course. The students were explicitly notified that the questionnaires pertained to their learning encounters during the term in the course. Following the elimination of invalid responses, 237 students constituted the final sample for quantitative scrutiny. The data analysis protocol encompassed several steps. Initially, a confirmatory factor analysis (CFA) was conducted to test the validity of the CRS and EPSSE questionnaires based on the hypothesized model. Subsequently, the questionnaires’ reliability was validated through the computation of Cronbach’s alpha coefficient. Pearson correlation analyses were then carried out to explore potential links between learners’ co-regulation and their self-efficacy in English public speaking. Structural equation modeling (SEM) was subsequently employed to probe the interplay across the components of CRS and EPSSE within the hypothesized model.

## Results

The Kolmogorov–Smirnov test was performed to assess the normality of the data. The findings verified the normal distribution of the data in this research, as indicated by the absolute kurtosis and absolute skewness values of all variables being below 3 (Marozzi, 2013).

### The CFA analysis of the CRS and EPSSE questionnaires

This study employed a unified CFA incorporating the items and factors in the questionnaires of CRS and EPSSE into a single analysis model. In the model, 27 items were retained, with 15 items for CRS and 12 items for EPSSE. The factor loadings, coefficients of Cronbach’s alpha, average variance extracted (AVE), and composite reliability (CR) for each survey factor’s items are presented in Table 1. The findings revealed that all the factor loadings exceeded the cutoff value (0.63–0.85, > 0.5) and were statistically significant, demonstrating the associations between the observed outcomes and the latent constructs. The Cronbach’s alpha (α) values for all factors (0.721–0.874) and the overall α value (0.955) in the two questionnaires suggested the reliability of these factors for measuring the two latent constructs. Furthermore, the AVE and CR were satisfactory for all the factors in the two questionnaires (AVE = 0.478–0.699, > 0.4; CR = 0.732 to 0.875, > 0.7) (Fornell and Larcker, 1981). The model analysis indicated good model fit parameters. The fit indices of the observed items demonstrate a high level of congruence with the specified model, with *χ*^2^/*df* = 2.382, *p* < 0.001, goodness of fit index (GFI) = 0.923, standardized root-mean-square residual (SRMR) = 0.051, comparative fit index (CFI) = 0.916, root mean square error of approximation (RMSEA) = 0.077, and RMSEA 90% CI = 0.069–0.084.

**Table 1:** CFA analysis of CRS and EPSSE.

Factors and items	Factor loading	S.E.	AVE	CR	α value
**Co-regulation strategies questionnaire**					
Co-planning (CP)			0.618	0.829	0.821
CP1	0.81	–			
CP2	0.828	0.071			
CP3	0.716	0.071			
Co-monitoring (CM)			0.478	0.732	0.721
CM1	0.765	–			
CM2	0.637	0.078			
CM3	0.664	0.075			
Co-evaluation (CE)			0.592	0.813	0.811
CE1	0.745	–			
CE2	0.764	0.087			
CE3	0.799	0.082			
Effort regulation (ER)			0.699	0.875	0.874
ER1	0.831	–			
ER2	0.839	0.065			
ER3	0.838	0.065			
Help seeking (HS)			0.532	0.773	0.776
HS2	0.681	–			
HS1	0.76	0.107			
HS3	0.744	0.102			
**English public speaking questionnaire**					
Language self-efficacy (LSE)			0.672	0.86	0.855
LSE1	0.851	–			
LSE2	0.814	0.063			
LSE3	0.792	0.069			
Delivery self-efficacy (DSE)			0.584	0.808	0.799
DSE1	0.822	–			
DSE2	0.736	0.075			
DSE3	0.732	0.075			
Topic self-efficacy (TSE)			0.612	0.826	0.826
TSE1	0.80	–			
TSE2	0.777	0.071			
TSE3	0.77	0.071			
Organization self-efficacy (OSE)			0.635	0.839	0.838
OSE1	0.797	–			
OSE2	0.809	0.072			
OSE3	0.784	0.065			

### Correlation analysis across the components of CRS and EPSSE

Initially, the presence of multicollinearity among the observed variables was evaluated through the calculation of Variance Inflated Factor (VIF) and item correlation coefficients. The findings revealed an absence of multicollinearity within the variables, as the highest VIF value was below 5, and the average VIF did not exceed 1 significantly (Cohen et al., 2003). Furthermore, the absolute values of the item correlation coefficients were all below 0.8, providing further evidence against the presence of multicollinearity among the variables.

Subsequently, the relationship between co-regulation and English public speaking self-efficacy was investigated using Pearson correlation analysis. Table 2 displays the correlation coefficients between the two questionnaires of CRS and EPSSE. The table illustrates significant positive correlations between all factors of the EPSSE and CRS (*r* = 0.402–0.821, *p* < 0.01).

**Table 2:** Descriptive analysis and correlation analysis among EPSSE and CRS questionnaires.

	*M*	*SD*	1	2	3	4	5	6	7	8
1. Organization self-efficacy	3.342	0.741	1							
2. Topic self-efficacy	3.404	0.697	0.800**	1						
3. Delivery self-efficacy	3.243	0.757	0.751**	0.790**	1					
4. Language self-efficacy	3.281	0.735	0.748**	0.857**	0.816**	1				
5. Help seeking	3.498	0.721	0.402**	0.393**	0.328**	0.410**	1			
6. Effort regulation	3.883	0.678	0.497**	0.498**	0.429**	0.446**	0.624**	1		
7. Co-evaluation	3.684	0.669	0.574**	0.544**	0.518**	0.517**	0.666**	0.803**	1	
8. Co-monitoring	3.693	0.669	0.476**	0.466**	0.423**	0.479**	0.699**	0.790**	0.783**	1
9. Co-planning	3.691	0.725	0.522**	0.542**	0.481**	0.523**	0.627**	0.781**	0.775**	0.821**

*p < 0.05; **p < 0.01.

### Path analysis

Based on the results of correlation analysis, a path model using SEM analysis was developed to explore the structural relationships across the components of co-regulation of English learners in collaborative oral presentations and those of learners’ self-efficacy in English public speaking. The results of the structural analysis using SEM demonstrated that the model effectively accounted for the data in the present study, with the fit parameters demonstrating a satisfactory model fit (*χ*^2^/*df* = 2.38, CFI = 0.916, TLI = 0.896, SRMR = 0.049, RMSEA = 0.076, RMSEA 90% CI = 0.069–0.084) (Kline, 2010).

**Figure 2:**
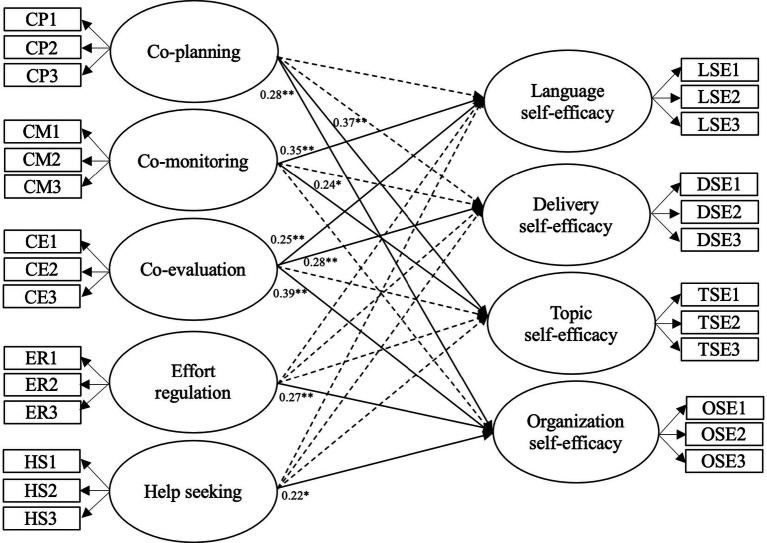
The final model of the effects co-regulation strategies on English public speaking self-efficacy. Solid lines indicate statistically significant effects, while dotted lines represent statistically insignificant effects; *p < 0.05; **p < 0.01.

The co-planning factor of CRS significantly contributed to explaining the variance in topic self-efficacy (*β* = 0.37, *p* < 0.01) and organization self-efficacy (*β* = 0.28, *p* < 0.01) among the EPSSE factors. The co-monitoring factor of CRS was a positive predictor for students’ language self-efficacy (*β* = 0.35, *p* < 0.01) and topic self-efficacy (*β* = 0.24, *p* < 0.05) within the EPSSE factors. The co-evaluation factor of CRS was found to have positive effects on learners’ language self-efficacy (*β* = 0.25, *p* < 0.01), delivery self-efficacy (*β* = 0.28, *p* < 0.01), and organization self-efficacy (*β* = 0.39, *p* < 0.01) among the EPSSE factors. The effort regulation factor and the help seeking factor in CRS were found to positively predict learners’ organization self-efficacy (*β* = 0.27, *p* < 0.01; *β* = 0.22, *p* < 0.05, respectively) among the EPSSE factors. Standardized estimates depicting the graphical path between variables are presented in Figure 2.

In conclusion, the results of the path analysis indicate significantly positive effects of co-planning strategy on self-efficacy in topic and organization in English public speaking. Co-monitoring strategy has direct and positive associations with language self-efficacy and topic self-efficacy in English public speaking. Co-evaluation strategy has direct and positive associations with language self-efficacy, delivery self-efficacy, and organization self-efficacy in English public speaking. Particularly, it is indicated that the effort regulation strategy and the help seeking strategy only play a direct and positive role on organization self-efficacy in English public speaking.

## Discussion

The CFA results confirm the reliability and validity of the CRS questionnaire, which categorizes co-regulation in collaborative oral presentations into five components: co-planning strategy, co-monitoring strategy, co-evaluation strategy, effort regulation strategy, and help seeking strategy. These findings are consistent with Su et al.’s (2023) study of the conceptualization of co-regulation strategies. Furthermore, the results validate the four components in the EPSSE questionnaire: self-efficacy beliefs in topic competence, language competence, organization competence, and delivery competence, aligning with Zhang and Ardasheva’s (2019) study, which defines public speaking self-efficacy as a multi-dimensional construct that directly relates to public speaking competence. Correlation analysis confirms the existence of positive associations between co-regulation and public speaking self-efficacy. The significant associations support previous research that highlights the relations between EFL learners’ use of co-regulation strategies and writing self-efficacy in collaborative writing contexts (Su et al., 2023). The present study further substantiates the findings by examining the associations between co-regulation and self-efficacy within the context of collaborative oral presentations.

The findings of this study demonstrate that co-planning significantly predicts learners’ self-efficacy in topic and organization for English public speaking. Co-planning, in this context, refers to the process of students collectively planning, setting goals, and dividing tasks for oral presentations. The results indicate that collaborative planning and goal setting have a positive impact on students’ belief in their ability to effectively select and maintain topics and to organize their oral presentation in a logical and clarified way. The relationship between co-planning and topic self-efficacy can be attributed to the fact that devising and dividing tasks among learners help alleviate the burden and self-imposed restrictions they may face when choosing and managing unfamiliar and challenging topics (Zhang and Ardasheva, 2019). Furthermore, in relation to co-planning and organization self-efficacy, it is important to note that oral presentations require not only proficiency in the English language, but also critical thinking ability, creativity in ideation, and logical organization (Lucas, 2010). Therefore, careful planning becomes essential for delivering well-organized speeches. This study emphasizes that when students collaborate in addressing the organizational aspects of the task within a group, their self-efficacy in organizing oral presentations is enhanced.

It was discovered that co-monitoring in English oral presentations plays a positive role on learners’ language and topic self-efficacy. In this study, co-monitoring refers to the ongoing management of students’ understanding, progress, and performance in oral presentation tasks. In collaborative work, EFL learners monitor different aspects of their cognition, beliefs, emotions, and motivational states in order to review, elaborate, revise, and improve the task responses of group members (Li et al., 2021). As a result, language, being the key cognitive component in oral presentations, becomes the focal point of co-monitoring and thus enhances belief in this aspect. Additionally, since monitoring the processes of the group task and understanding the requirements of the task helps learners establish common task comprehension and negotiate task goals for the achievement of consensus (Malmberg et al., 2017), it can be assumed that selecting and maintaining a topic in oral presentations would be one of the areas of focus when interpreting task requirements. Following this principle, students’ self-efficacy in topic can be enhanced.

Given the significance of the co-monitoring strategy in promoting public speaking self-efficacy as established in this study, as well as its crucial role in fostering effective collaborative learning (Volet et al., 2009), practitioners in EFL speaking instruction need to set effective mechanisms to enhance learners’ regulatory behaviors in co-monitoring collaborative oral presentation activities.

It is suggested that co-evaluation in English oral presentations is the most significant co-regulation strategy that influences learners’ self-efficacy in English public speaking. This strategy has an impact on three dimensions of English public speaking self-efficacy: language self-efficacy, delivery self-efficacy, and organization self-efficacy. This result aligns with a previous study conducted by Al-Issa and Al-Qubtan (2010), which found that co-evaluation was widely appreciated by learners and effectively fostered their self-efficacy and intrinsic motivation. The present study expands on these findings by investigating the specific dimensions of self-efficacy and the context of collaborative oral presentations. In this context, co-evaluation refers to the way students assess their group’s performance in presentation tasks and is recognized as one crucial co-regulation strategy to influence the collaborative activities among learners (Järvelä and Hadwin, 2013). Learners who actively engage in evaluating their performance tend to focus on both the end product and the process itself (Zimmerman, 2008). In the case of collaborative oral presentations, students choose to co-evaluate the product of their speech, including aspects of linguistic accuracy, fluency, and logical sequencing, as well as the process of their performance, such as emotional and physical control during delivery. Consequently, their self-efficacy in these areas is more likely to be enhanced. This finding underscores the importance of providing necessary scaffolding to help learners actively and effectively review and evaluate their performance in collaborative oral presentations, for the purpose of promoting their self-efficacy in English public speaking.

The current study discovered a significant relationship between effort regulation and learners’ organization self-efficacy in English public speaking. In the current study, effort regulation refers to students’ ability to persist when faced with obstacles or challenges during collaborative oral presentation tasks. Effort regulation in collaborative learning requires students to regulate both themselves and their group members as they work together, exerting influence on one another. The results indicate that students who can effectively regulate their effort when encountering difficulties in completing oral presentation tasks are more likely to possess self-efficacy in organizing their public speaking performance with logic and clarity. Previous research has indicated that self-efficacy has a noteworthy influence on effort regulation in academic performance (Honicke and Broadbent, 2016). However, our study suggests that the impact may be reversed in the context of collaborative oral presentations in which effort regulation has a noteworthy influence on self-efficacy, indicating a different direction of influence. Consequently, it is worth investigating the reciprocal relationship between effort regulation and self-efficacy in collaborative work, which could be a potential area of focus for future research in light of the finding of this study.

The results have also indicated that help-seeking significantly predicts learners’ self-efficacy in organizing themselves for English public speaking. This implies that when students seek assistance from others to overcome challenges in collaborative oral presentations, they develop greater self-efficacy in organizing their public speaking in a logical and clear manner. These findings confirm previous claims that the anxiety experienced by EFL speakers during oral presentations is due to a sense of helplessness (Hsu, 2012), and that students who avoid seeking help tend to experience higher levels of anxiety and lower motivation (Karabenick, 2003). Additionally, our findings highlight the positive impact of receiving assistance during collaborative oral presentations on students’ self-efficacy in organizing their public speeches. This finding is reminiscent of a study conducted by Chou (2011), wherein it was discovered that groups encountered challenges in summarizing the key points in the concluding section of their oral presentations. Nonetheless, Chou’s study did not specifically examine the concept of seeking assistance, thereby impeding the exploration of the potential impact of this strategy on students’ self-efficacy in structuring their organization with coherence and cogency.

## Conclusion

The current study examines the impact of co-regulation strategies in collaborative oral presentations on the English public speaking self-efficacy of EFL learners. The findings emphasize the positive influence of the five components of co-regulation in explaining different aspects of learners’ English public speaking self-efficacy. This research contributes to the existing literature on English public speaking and self-efficacy by specifically investigating the role of co-regulation strategies within the context of collaborative oral presentations. Furthermore, this study has confirmed the effectiveness of the co-regulation questionnaire in assessing learners’ use of co-regulation strategies during collaborative oral learning activities. This questionnaire serves as a critical tool for evaluating the implementation of co-regulation by students.

These findings also carry significant implications for English public speaking pedagogy. Successful team learning necessitates more than simply assigning group assignments; university faculty must provide explicit instruction on how to work cooperatively and effectively leverage group work (Yang, 2010). The current study proposes that EFL instructors and curriculum designers should explore efficient methods to incorporate teaching with effective strategies of co-planning, co-monitoring, co-evaluation, effort regulation, and help-seeking in collaborative oral learning. Firstly, given the substantial role of co-evaluation found in this study, it is imperative for instructors to establish a responsive presence in order to support students in extracting valuable insights from the questions posed in their group discussions (Seau and Azman, 2022). For this purpose, the utilization of computer-assisted language learning techniques could be considered. One approach that instructors can employ is to create tasks that encourage students to engage in meaningful interactions, enabling them to analyze, evaluate, and assess their performance and the information they have acquired. The instructor’s active involvement in facilitating such activities can further foster the cultivation of a reflective and analytical mindset in group learning (Aljohani, 2024). Secondly, in terms of the impact of co-planning and co-monitoring on public speaking self-efficacy, it is the instructor’s duty to guide students in developing effective planning strategies for each phase of public speaking preparation. This includes topic selection, script writing, rehearsal, and the final performance. It is essential for students to learn how to establish timelines for each phase in order to effectively monitor and regulate their learning progress (Li et al., 2021). Thirdly, the impact of effort regulation and help seeking strategies on public speaking self-efficacy necessitates the supervision of students’ efforts and engagement by instructors. Instructors should also motivate group members to confront challenges and overcome obstacles in order to enhance effort regulation (Sungur, 2007). Additionally, it is advisable to encourage students to facilitate their peers’ learning by providing explanations rather than simply giving answers when asked for help (Webb et al., 2013) in order to promote the effectiveness of help seeking strategy.

This research study presents a number of limitations. Firstly, the data utilized in this study were restricted to grade-one EFL learners from a singular university, thus yielding a relatively small sample size. Consequently, the applicability of the findings is inherently restricted to this specific cohort of students. Secondly, this study is limited by its reliance on questionnaire survey results provided by the learners themselves. Consequently, the extent to which learners responded to teachers’ feedback and the impact of such feedback on learners’ performance in public speaking remains unknown.

Based on the findings and limitations of this study, future studies can further explore the following issues. First, considering the significant roles of co-regulation on self-efficacy found in this study, it is important to conduct a more in-depth examination of these two constructs in future research. Specifically, since it is suggested that co-regulation and self-efficacy may contribute to learner engagement in collaborative work (Volet et al., 2009), future research can be conducted to include learner engagement in relation to examine its influence by co-regulation strategies and self-efficacy for a broader understanding of co-regulation in collaborative language learning. The role of self-efficacy as a mediator in the relationship between co-regulation and learner engagement, as well as their reciprocal relationship, can be considered as key focal points. Second, conducting an examination of subsequent grade levels, where students have had a longer exposure to EFL public speaking, across multiple universities may reveal different patterns and provide a more comprehensive understanding. Lastly, in order to enhance the rigor and comprehensiveness of future research, it is recommended to integrate qualitative analysis to explore the influence of teachers’ feedback on learners’ co-regulation and to conduct surveys on the outcomes of EFL public speaking. This approach would contribute to a more robust comprehension of the phenomenon being investigated.

## Data Availability

The raw data supporting the conclusions of this article will be made available by the authors, without undue reservation.

## Appendix

### Appendix A: English public speaking self-efficacy questionnaire

**Language self-efficacy**

1. When giving an English speech in public, I can speak with accuracy.
2. When giving an English speech in public, I can speak with fluency.
3. When giving an English speech in public on an unfamiliar or difficult topic, I can speak effectively.

**Delivery self-efficacy**

4. When giving an English speech in public, I can speak with emotion.
5. I can give an English speech in public when I am very nervous.
6. When giving an English speech in public, I can speak with confidence.

**Topic self-efficacy**

7. When giving an English speech in public, I can organize my speech so that the conclusion flows logically from what was previously said.
8. When giving an English speech in public, I can use appropriate language (e.g., vocabulary, grammatical structures) to address different topics.
9. When giving an English speech in public, I can make my central idea clear to the audience.

**Organization self-efficacy**

10. When giving an English speech in public, I can use inductive techniques (proceeding from details to generalization/argument) to structure a speech.
11. When giving an English speech in public, I can use deductive techniques (proceeding from generalization/argument) to structure a speech.
12. When giving an English speech in public, I can use the conclusion to restate my main points.

### Appendix B: Co-regulation strategy questionnaire

**Co-planning**

1. When working in our English presentation group, I try to make sure we set learning goals and allocate time for various activities.
2. When working in our English presentation group, I try to make an outline for our presentation.
3. When working in our English presentation group, I try to allocate the tasks to group members.

**Co-monitoring**

4. When working in our English presentation group, I often ask others what they think about the presentation topic.
5. When we are doing the collaborative English presentation tasks, I make up questions to ask our group members to help find out whether we have understood the work.
6. When our group has different ideas, I try to help us reach an agreement.

**Co-evaluation**

7. I carefully read the comments from my classmates and put forward suggestions for promoting my group’s English presentation.
8. I carefully assess my group’s presentation according to the scoring rubrics and put forward suggestions for improving the presentation quality.
9. I often make suggestions for improving my group’s presentation quality according to the teacher’s feedback.

**Effort regulation**

10. When the English presentation task is difficult, I do not give up.
11. When working in our English presentation group, I work hard to do well even if I do not like what we are doing.
12. When working in our English presentation group and the topic is not interesting, I manage to keep on contributing my ideas until we finish the task.

**Help seeking**

13. When working in our English presentation group, I often turn to my team members for help.
14. When working in our English presentation group, I often ask my team members to explain concepts I do not understand well.
15. When working in our English presentation group, I often ask my team members to check or revise what I provide.
